# Atypical Response Properties of the Auditory Cortex of Awake MECP2-Overexpressing Mice

**DOI:** 10.3389/fnins.2019.00439

**Published:** 2019-05-07

**Authors:** Chang Zhou, Sumei Yan, Shaowen Qian, Zhaoqun Wang, Zhiyue Shi, Ying Xiong, Yi Zhou

**Affiliations:** Department of Neurobiology, School of Basic Medical Sciences, Army Medical University, Chongqing, China

**Keywords:** MECP2 duplication syndrome, auditory cortex, electrophysiology, autism, tonal receptive field

## Abstract

Methyl-CpG binding protein 2 (MECP2) is a gene associated with DNA methylation and has been found to be important for maintaining brain function. In humans, overexpression of MECP2 can cause a severe developmental disorder known as MECP2 duplication syndrome. However, it is still unclear whether MECP2 overexpression also causes auditory abnormalities, which are common in people with autism. MECP2-TG is a mouse model of MECP2 duplication syndrome and has been widely used for research on social difficulty and other autism-like disorders. In this study, we used a combination of multiple electrophysiological techniques to document the response properties of the auditory cortex of awake MECP2-TG mice. Our results showed that while the auditory brainstem responses are similar, cortical activity patterns including local field potentials (LFPs), multiunit activity (MUA), and single-neuron responses differ between MECP2-TG and wild-type (WT) mice. At the single-neuron level, the spike waveform of fast-spiking (FS) neurons from MECP2-TG mice is different from that of WT mice, as reflected by reduced peak/trough ratios in the transgenic mice. Both regular-spiking (RS) and FS neurons exhibited atypical response properties in MECP2-TG mice compared with WT mice, such as prolonged latency and an elevated intensity threshold; furthermore, regarding the response strength to different stimuli, MECP2-TG mice exhibited stronger responses to noise than to pure tone, while this pattern was not observed in WT mice. Our findings suggest that MECP2 overexpression can cause the auditory cortex to have atypical response properties, an implication that could be helpful for further understanding the nature of auditory deficits in autism.

## Introduction

Autism spectrum disorder (ASD) comprises a variety of neurological disorders characterized by delayed language learning and impairments in social communication (Rapin and Dunn, 2003; Siegal and Blades, 2003; Cardy et al., 2008). Methyl-CpG-binding protein 2 (MECP2), encoded by the X-linked transcriptional repressor gene Mecp2, is critical for normal brain development (Qiu, 2017). Previous studies have demonstrated that either loss or duplication of MECP2 results in neurodevelopmental disorders characterized by multiple autistic behaviors (Amir et al., 1999; Daniela et al., 2006). Loss-of-function mutations in MECP2 can cause Rett syndrome (RTT) (Chahrour and Zoghbi, 2007), while gain-of-function mutations result in MECP2 duplication syndrome (Esch et al., 2005; Ramocki et al., 2010). The auditory sense receives messages from the outside environment and plays a critical role in language and social communication. Previous studies indicate that patients with ASD exhibit different degrees of aberrant reactivity to auditory stimuli, especially noise stimuli (Siegal and Blades, 2003; O’Connor, 2012), suggesting dysfunction in auditory perception. Experimental evidence has shown that the auditory brainstem responses (ABRs) of patients with ASD to auditory stimuli are largely normal (Bader et al., 1989; Kálmánchey, 1990), suggesting that higher regions in the auditory pathway are an underlying cause of abnormal auditory perception in ASD. In addition, previous studies have also revealed that patients with Rett syndrome exhibit degraded auditory cortex responses, including delayed onset and peak latencies, reduced local field potential (LFP) amplitudes and weakened multiunit responses (Bader et al., 1989; Stauder et al., 2006). However, it remains largely unclear how MECP2 overexpression affects the auditory cortex in mouse models of MECP2 duplication syndrome.

To explore the potential change in fundamental properties of the auditory cortex caused by MECP2 overexpression, we investigated a transgenic mouse line called MECP2-TG, which overexpresses MECP2 due to insertion of the human MECP2 gene. MECP2-TG mice exhibit many symptoms similar to those of MECP2 duplication syndrome in humans, including repetitive stereotypes and abnormal communication and social behavior (Collins et al., 2004). We first measured the ABRs in both MECP2-TG mice and wild-type (WT) littermates to examine changes in basic hearing. Multiunit activity (MUA) recordings and single-cell recordings were performed *in vivo* to investigate the fundamental properties of the auditory cortex, such as tonotopy, threshold map and spike activities. Single-cell recordings were then clustered into regular-spiking (RS) and fast-spiking (FS) cells based on the bimodal distribution of spike waveforms. Tonal receptive fields (TRFs) were compared in detail between different cells in the two animal groups. A stronger response to white noise stimulation than to pure tone stimulation was found in MECP2-TG mice but not WT controls. Our findings suggest that MECP2 overexpression can cause the auditory cortex to have atypical response properties, an implication that could be helpful for further understanding the nature of auditory deficits in autism.

## Materials and Methods

### Animals

Adult male mice (12–16 weeks old, weighing 27–34 g) were used in this study. The mice were maintained in standard housing on a 12 h light/12 h dark cycle. All the male MECP2-TG mice and WT littermate mice were on an FVB/N background. The experimenters were blinded to the genotypes of the animals. All experimental procedures used in this study were approved by the Animal Care and Use Committee of Army/Third Military Medical University (SYXK-PLA-20120031).

### *In vivo* ABR Recording

The mice were anesthetized with ketamine (45 mg/kg) and xylazine (6.4 mg/kg) by intraperitoneal injection. The experiments were performed in a double-shielded sound-attenuating booth (Shenyang Sound-attenuating Booth Factory, China). Three metal electrodes were inserted subcutaneously into the left mastoid process, calvarium and right hindlimb. A free-field magnetic speaker (MF1, TDT Inc., United States) was positioned 10 cm away from the left ear. The speaker was driven by a stereo power amplifier (RZ6, TDT Inc., United States). Click sounds (0.1 ms duration per click, 10 ms interval) of various intensities [0–30 dB sound pressure level (SPL) at 5 dB intervals and 30–70 dB SPL at 10 dB intervals] were delivered to the left ear for a total of 1024 trials. The location of peaks in ABR data were determined online by SigGenRZ (TDT Inc., United States) and stored offline for statistical analysis.

### Animal Preparation and Tone Stimulation

Mice were anesthetized with ketamine (45 mg/kg) and xylazine (6.4 mg/kg) by intraperitoneal injection. The experiments were performed in a double-shielded sound-attenuating booth. After the skin was removed and the calvarium was cleaned, a customized apparatus was used to fix the head in place. Intramuscular injection of lidocaine was used to further relieve the pain. A craniotomy was performed to expose the right auditory cortex. The ear canal on the same side was plugged with a cotton ball. A free-field magnetic speaker was positioned 5 cm from the contralateral ear (left ear). The speaker was driven by a stereo power amplifier (SA1, TDT Inc., United States) and calibrated using a 1/4-pressure prepolarized condenser microphone setup (377A01 microphone, 426B03 preamplifier, 480E09 signal conditioner, Piezotronics Inc., United States). The captured signals were sampled at 1 MHz/s with a high-speed data acquisition (DAQ) board (PCI-6251, National Instruments, United States). Customized LabVIEW programs were used for calibration and sound generation with a distortion of ± 0.6 dB SPL at 70 dB SPL (0.5–64 kHz). We mapped the tonotopy of the cortex through extracellular recordings with parylene-coated tungsten electrodes (0.1 MΩ, WPI Inc., United States) 400–600 μm below the pia. Pure tones (35 ms duration, 5 ms ramp) of various frequencies (0.5–64 kHz, 0.2-octave intervals) and intensities (0–70 dB SPL at 10 dB intervals) were generated by custom software (LabVIEW, National Instruments), and 288 testing stimuli were presented in a pseudorandom sequence (150 ms intervals). The primary auditory cortex was identified by its anatomical location in the mouse brain (1.5–3.5 mm from the bregma) and by its patterned tonotopy as previously described (Wei et al., 2012). The cortical surface was covered with warm (37°C) artificial cerebrospinal fluid [ACSF, containing (in mM) 124 NaCl, 1.2 NaH_2_PO_4_, 2.5 KCl, 25 NaHCO_3_, 20 glucose, 2 CaCl_2_, and 1 MgCl_2_]. After mapping, the opening was temporarily sealed with silicone rubber (Body Double, Smooth on Inc., United States), and the mouse was returned to a clean cage to recover for 1 day.

### *In vivo* LFP Recording and Extracellular Multiunit Recording

After mapping, LFP recording and extracellular multiunit recording were performed with parylene-coated tungsten electrodes (0.1 MΩ, WPI Inc., United States) in awake mice. The electrode was inserted into the cortex vertically with a micromanipulator (MC1000e and 7600, Siskiyou, United States) to a depth of 400–600 μm below the pia. Pure tones (7000 Hz, 70 dB SPL, 35 ms duration, 5 ms ramp) or white noise (70 dB SPL, 35 ms duration, 5 ms ramp) were delivered to the left ear at least 30 times. Neural signals were amplified and collected by a TDT System 3 (LFP signals – gain: 5000, sampling rate: 50 kHz; extracellular multiunit signals – gain: 20000, sampling rate: 50 kHz; TDT Inc., United States). For LFP recording, the high-pass filter was set at 1 Hz, and the low-pass filter was set at 300 Hz for neural activity. The LFP amplitude was calculated as the difference between the peak amplitude and the averaged amplitude in a 10 ms window before the onset of stimulation. The onset latency was measured based on a threshold set at three times the standard deviation above the baseline. For the recording of extracellular multiunit signals, the high-pass filter was set at 300 Hz, and the low-pass filter was set at 3000 Hz for spike activity. The threshold for spike detection was set at three times the standard deviation above the baseline. The data were analyzed online and stored for offline analysis with BrainWare (TDT Inc., United States).

### Single-Unit Activity (SUA) Recordings

*In vivo* patch-clamp recording and extracellular microwire recording were used to obtain SUA from awake mice. *In vivo* loose-patch-clamp recording was performed as previously described (Zhou et al., 2017). For extracellular microwire recording, customized 4-channel microwire electrodes with an impedance of approximately 0.5 MΩ were used. The electrode was inserted into the cortex vertically with a micromanipulator (MC1000e and 7600, Siskiyou, United States) in a similar manner to the tungsten electrode used for mapping. Pure tones (0.5–64 kHz at 0.2-octave intervals; 0–70 dB in 10 dB steps) (35 ms duration, 5 ms ramp) were delivered to the left ear at least 3 times (at an interstimulus interval of 150 ms) to obtain the TRF. Data were recorded using a Plexon Omniplex system (Plexon Inc., United States) and further analyzed offline (Offline Sorter 4.0, Plexon, United States). The peak latency was the lag between the stimulus onset and the peak of the firing rate. The half-peak duration of the peri-stimulus spike time histogram (PSTH) was calculated as the lag between two points where the PSTH crossed a threshold set at half its maximum amplitude (“peak”). The intensity selectivity index was calculated as previously described (Li et al., 2015). The average activity was calculated over a 50 ms time window after the onset of sound stimulation.

### Statistical Analysis

All data analysis was performed using customized codes in MATLAB (Mathworks Inc., United States) that blinds the researcher to the experimental groups. Experimental data are presented as the mean ± SEM unless specified. Statistical analyses were conducted using SPSS 16.0 software (SPSS, United States). The significance of differences between groups was calculated by Student’s *t*-test. Two-sided *p*-values were calculated, and the graphs are marked with one star “*” to indicate *p* < 0.05, two stars “**” to indicate *p* < 0.01, or three stars “***” to indicate *p* < 0.001, all of which were considered statistically significant.

## Results

### ABRs in MECP2-TG Mice Are Normal

**FIGURE 1:**
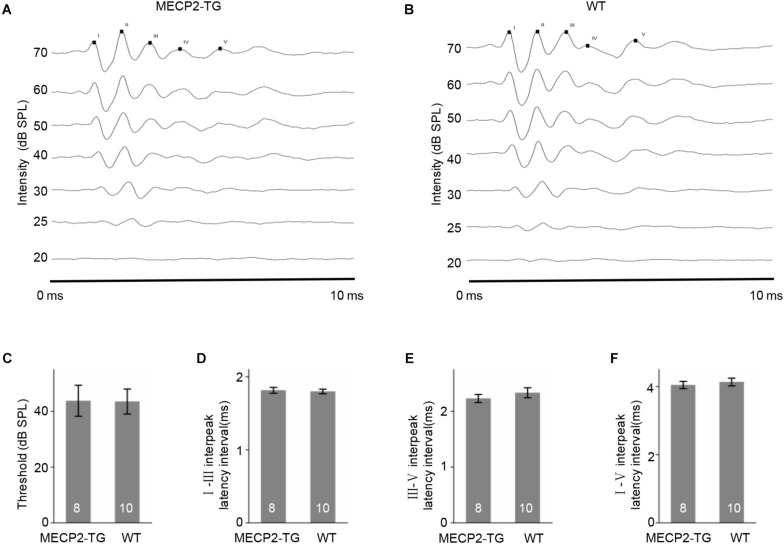
**(A,B)** Examples of auditory brainstem responses (ABRs) in MECP2-TG mice and wild-type (WT) mice, respectively. **(C)** The threshold of ABRs in MECP2-TG mice and WT mice, respectively. *t*-test, mean ± standard error of the mean (SEM). No significant difference, *p* = 0.97, *t*-test. **(D)** The I–III inter-peak latency interval of ABR in MECP2-TG mice and WT mice, respectively. *t*-test, mean ± SEM. No significant difference, *p* = 0.77, *t*-test. **(E)** The III–V inter-peak latency interval of ABR in MECP2-TG mice and WT mice, respectively. *t*-test, mean ± SEM. No significant difference, *p* = 0.40, *t*-test. **(F)** The I–V inter-peak latency interval of ABR in MECP2-TG mice and WT mice, respectively. *t*-test, mean ± SEM. No significant difference, *p* = 0.58, *t*-test.

To examine whether MECP2 overexpression causes fundamental hearing difficulties, we recorded ABRs from MECP2-TG and WT mice (see section Materials and Methods for details). Figures 1A,B showed two representative ABR results (10–70 dB) and Supplementary Figure S1 showed more raw traces with marked peaks at 70 dB. No significant difference was found between the thresholds for MECP2-TG mice and WT mice (Figure 1C). The temporal properties of ABRs, such as the I–III inter-peak latency interval, III–V inter-peak latency interval, and I–V inter-peak latency interval, also showed no significant difference between the two animal groups (Figures 1D–F). These results suggest that MECP2-TG mice have no major hearing abnormalities in early hearing pathway.

### An Increased Threshold Was Found in MECP2-TG Mice

**FIGURE 2:**
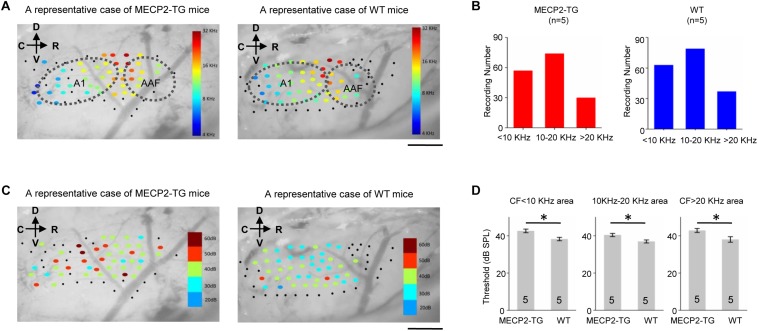
**(A)** A representative case of the characteristic frequency (CF) distribution in the auditory cortex in MECP2-TG mice and wild-type (WT) mice, respectively. Black dots indicate that there was no response at the recording site. Scale bar, 0.5 mm. **(B)** Distribution of characteristic frequency of 168 recording sites from MECP2-TG mice (*n* = 5) and 174 recording sites from WT mice (*n* = 5). **(C)** Example cortical threshold map from the auditory cortex of MECP2-TG mice and WT mice. Black dots indicate that there was no response at the recording site. Scale bar, 0.5 mm. **(D)** Average intensity threshold at CF of low-frequency regions, middle-frequency regions and high-frequency regions in MECP2-TG mice (*n* = 5) and WT mice (*n* = 5). *p < 0.05, *t*-test, mean ± standard error of the mean (SEM).

Characteristic frequency (CF) distribution and threshold reflect fundamental properties of hearing function and related structure in the auditory cortex. We used extracellular recordings to investigate the tonotopy of the cortex and generated cortical threshold in awake MECP2-TG mice and WT mice. Figure 2A shows representative cases of tonotopy. The CF distribution and frequency gradient are clear and largely similar in both animal groups. On the other hand, the cortical threshold map that show the distribution of thresholds are different between MECP2-TG mice and WT mice (Figure 2C). Statistical results showed a significantly higher threshold in MECP2-TG mice than in WT mice in low-frequency, middle-frequency, and high-frequency areas (Figure 2D). This difference suggests that the transgenic mice could have hearing abnormalities related to the auditory cortex, gradually arising in the ascending auditory pathway.

### MECP2 Overexpression Alters the Spike Waveforms of FS but Not RS Cells

The waveform of spikes can be used as a standard to group single-cell recordings into different subtypes   (Levy and Reyes, 2012).

**FIGURE 3:**
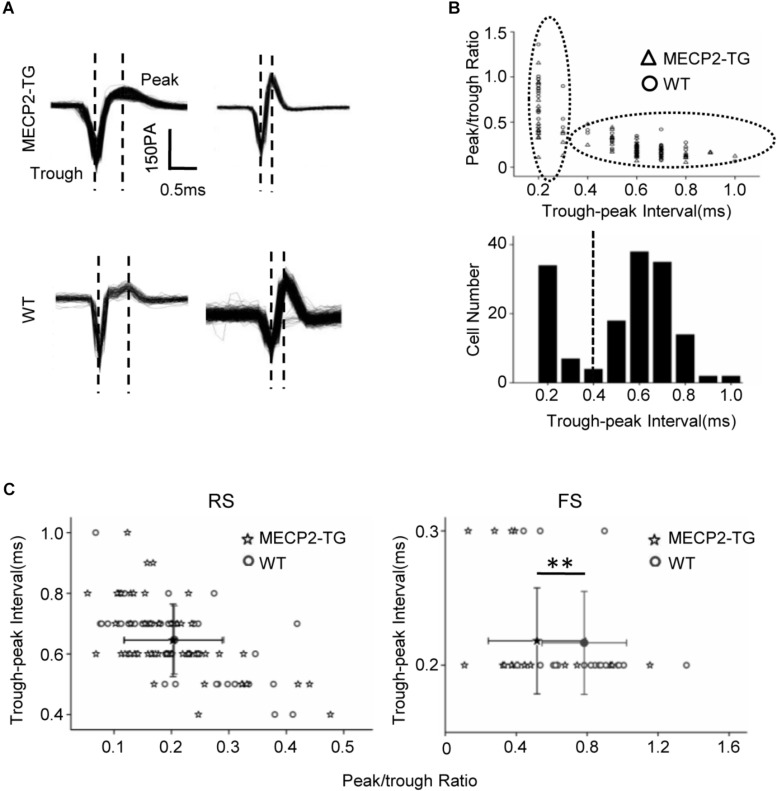
**(A)** Representative spike waveforms. Dashed lines indicate the trough and peak of the spike waveform. **(B)** Upper panel, scatter plot of peak/trough ratios and trough-peak intervals of spike waveforms from all recorded neurons. Bottom panel, histogram of the peak-trough intervals recorded from all recorded neurons. **(C)** Scatter plot of the peak/trough ratio and trough-peak interval of spike waveforms from regular-spiking neurons and fast-spiking neurons, respectively. Peak/trough ratio: **p < 0.01; *t*-test, mean ± standard deviation.

Figure 3A shows representative traces recorded from MECP2-TG and WT mice. The peak-trough interval and peak/trough ratio were measured to quantify the shape of spike waveforms (Figure 3B). A bimodal distribution of peak-trough intervals was found, and 0.4 ms was chosen as a cut off to separate RS neurons (putatively excitatory pyramidal cells) and FS neurons (putatively inhibitory basket and chandelier cells). In addition, the peak/trough ratio of RS neurons from two animals showed no significant difference, but FS neurons of MECP2-TG mice had a lower peak/trough ratio than WT neurons had (Figure 3C). This finding shows that overexpression of MECP2 had a larger influence on FS neurons than on RS neurons in MECP2-TG mice. The abnormal waveform could be a result of altered intracellular dynamics (such as ion channels) related to the overexpression of the MECP2 gene. Normal RS and abnormal FS cells could further cause an imbalance between excitation and inhibition in the processing of auditory information.

### MECP2 Duplication Shapes the TRFs of Both RS and FS Neurons

**FIGURE 4:**
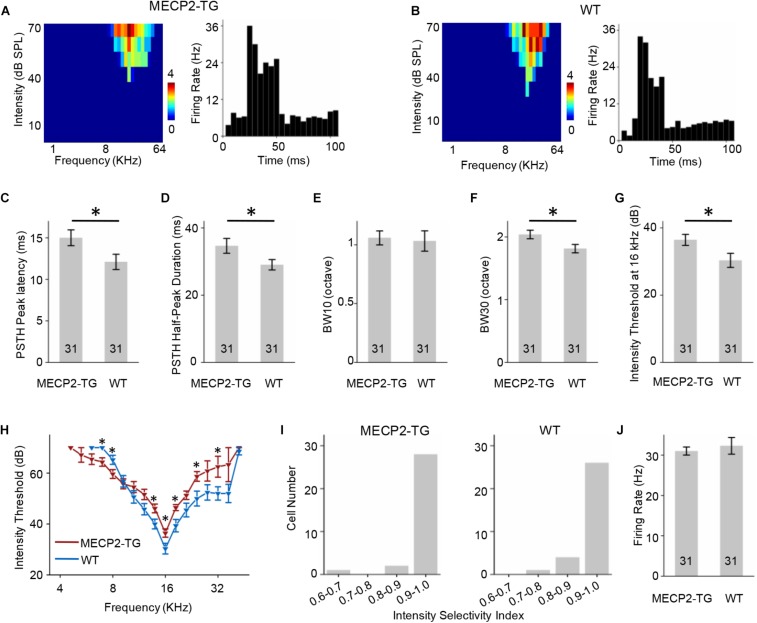
**(A,B)** Left, representative tonal receptive field (TRF) of a regular-spiking (RS) neuron. Right, representative peri-stimulus spike time histogram (PSTH) of an RS neuron. **(C)** Averaged peak latency of RS neurons recorded from MECP2-TG mice and wild-type (WT) mice, respectively. *p < 0.05, *t*-test, mean ± standard error of the mean (SEM). **(D)** Averaged duration at the half-maximum level in the PSTHs of RS neurons recorded from MECP2-TG mice and WT mice, respectively. *p < 0.05, *t*-test, mean ± SEM. **(E,F)** Average tuning bandwidth of TRFs at 10 and 30 dB above the intensity threshold of RS neurons recorded from MECP2-TG mice and WT mice. *p < 0.05, *t*-test, mean ± SEM. **(G)** Average intensity threshold at characteristic frequency (CF, 16 kHz) in RS neurons recorded from MECP2-TG mice and WT mice, respectively. *p < 0.05, *t*-test, mean ± SEM. **(H)** Average intensity threshold for each frequency tested all along the tuning curve in RS neurons recorded from MECP2-TG mice and WT mice, respectively. **(I)** Distribution of intensity selectivity indices of RS neurons recorded from MECP2-TG mice and WT mice, respectively. Bin size = 0.1. **(J)** Averaged activity of RS neurons to best frequency (BF) ± 0.2 octave at 70 dB. *t*-test, mean ± SEM.

A tonal receptive field is a basic property of auditory neurons. Different intensities (0–70 dB in 10 dB steps) and frequencies (0.5–64 kHz in 0.2-octave steps) of pure tone stimulation were used to determine the TRF properties of RS neurons from MECP2-TG and WT mice. For better demonstration, Figure 4 showed the results of CF around 16 KHz and other data were provided in Supplementary Figure S2 as a function of CF. To compare the response temporal profiles, we generated spike TRFs and PSTHs from spike responses (Figures 4A,B). We found that the peak latency in response to tone stimulation was significantly delayed in MECP2-TG mice compared to WT mice (Figure 4C). Then, we measured the half-peak durations of the PSTHs to examine the temporal response properties. The half-peak durations of RS neurons from MECP2-TG mice were broader than those of RS neurons from WT mice (Figure 4D). To quantify the frequency selectivity of RS neurons, we measured the bandwidth of spike TRF at intensities 10 and 30 dB above the threshold. RS neurons from MECP2-TG mice exhibited broader bandwidth at BW10 and BW30 than those from WT (Figures 4E,F), suggesting that the former neurons have lower frequency selectivity than the latter at high intensity levels. The intensity threshold of RS neurons from MECP2-TG mice was significantly higher than that of RS neurons from WT mice (Figures 4G,H). To quantify the intensity tuning, we calculated an intensity selectivity index (ISI) for the CF-tone-evoked responses of each recorded RS neuron. The majority of recorded RS neurons had monotonically increasing response-versus-intensity functions for both animal groups (Figure 4I). The firing rate in response to 70 dB pure tones [best frequency (BF) ± 0.2 octave] showed no significant difference between the two mouse strains (Figure 4J).

**FIGURE 5:**
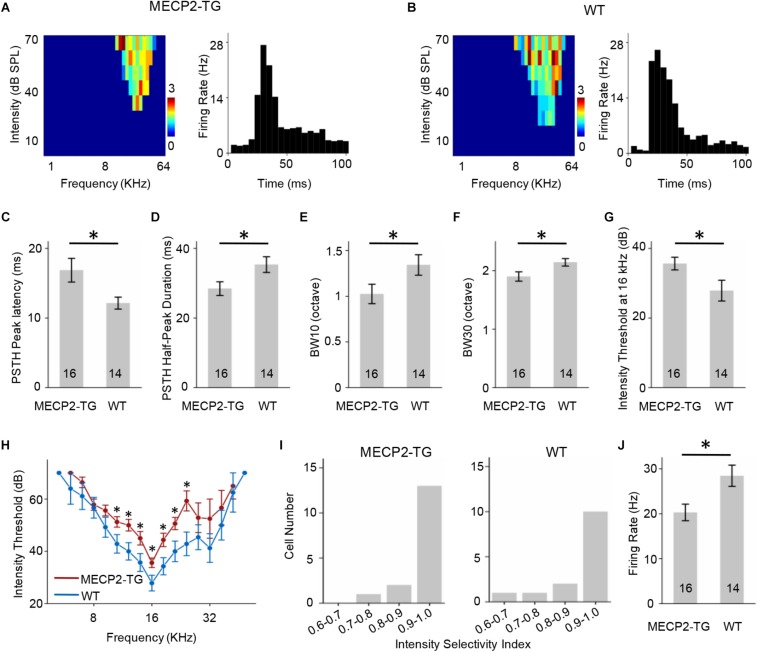
**(A,B)** Left, representative tonal receptive fields (TRFs) of fast-spiking (FS) neurons. Right, representative peri-stimulus spike time histograms (PSTHs) of FS neurons. **(C)** Averaged peak latency of FS neurons recorded from MECP2-TG mice and wild-type (WT) mice. *p < 0.05, *t*-test, mean ± standard error of the mean (SEM). **(D)** Averaged duration at the half-maximum level on the PSTHs of FS neurons recorded from MECP2-TG mice and WT mice. *p < 0.05, *t*-test, mean ± SEM. **(E,F)** Average tuning bandwidth of TRFs at 10 dB and 30 dB above the intensity threshold in FS neurons recorded from MECP2-TG mice and WT mice. *p < 0.05, *t*-test, mean ± SEM. **(G)** Average intensity threshold at the characteristic frequency (CF, 16 kHz) in FS neurons recorded from MECP2-TG mice and WT mice. *p < 0.05, *t*-test, mean ± SEM. **(H)** Average intensity threshold for each frequency tested all along the tuning curve in FS neurons recorded from MECP2-TG mice and WT mice. **(I)** Distribution of intensity selectivity indices of FS neurons recorded from MECP2-TG mice and WT mice. Bin size = 0.1. **(J)** Averaged activity of FS neurons in response to the best frequency (BF) ± 0.2 octave at 70 dB. *p < 0.05, *t*-test, mean ± SEM.

Similar to Figures 4, 5 showed the results of FS neurons with CF around 16 KHz and other data were provided in Supplementary Figure S3 as a function of CF. The FS neurons from MECP2-TG and WT mice both exhibited V-shaped TRFs (Figures 5A,B). The peak of the PSTH for the FS neurons from MECP2-TG mice appeared later than that of the PSTH for the FS neurons from WT mice (Figure 5C). The half-peak durations of FS neurons from MECP2-TG mice were narrower than those of FS neurons from WT mice (Figure 5D). To quantify the frequency selectivity of FS neurons from MECP2-TG and WT mice, we measured BW10 and BW30 for FS neurons. The FS neurons from MECP2-TG mice exhibited a narrower bandwidth than WT mice at both BW10 and BW30 (Figures 5E,F). The intensity threshold of FS neurons from MECP2-TG mice was significantly higher than that of FS neurons from WT mice (Figures 5G,H). The majority of recorded FS neurons in each animal group had monotonically increasing response-versus-intensity functions (Figure 5I). The firing rate of FS neurons to 70 dB SPL pure tones (BF ± 0.2 octave) was lower in MECP2-TG mice than in WT mice (Figure 5J). Overall, these results are different from the results obtained for RS neurons, suggesting that the overexpression of MECP2 plays a distinct role in shaping the TRF of cortical neurons in MECP2-TG mice.

### MECP2 Duplication Increases Cortical Responses to Noise

**FIGURE 6:**
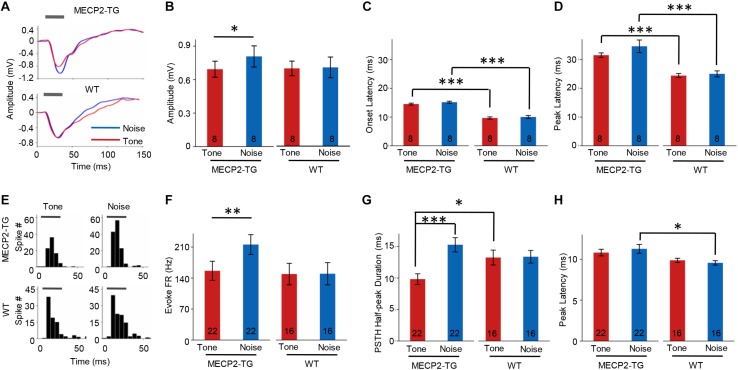
**(A)** Representative traces of local field potentials (LFPs) recorded from MECP2-TG and wild-type (WT) mice. The gray bar indicates the occurrence of stimuli (red line: tone stimulus; blue line: noise stimulus). **(B)** Averaged amplitude of LFPs recorded from MECP2-TG mice and WT mice. *p < 0.05, paired *t*-test, mean ± standard error of the mean (SEM; red: tone stimulus; blue: noise stimulus). **(C)** Averaged onset latency of LFPs recorded from MECP2-TG mice and WT mice. ***p < 0.001, *t*-test, mean ± SEM (red: tone stimulus; blue: noise stimulus). **(D)** Averaged peak latency of LFPs recorded from MECP2-TG mice and WT mice. ***p < 0.001, *t*-test, mean ± SEM (red: tone stimulus; blue: noise stimulus). **(E)** Representative peri-stimulus spike timing histograms (PSTHs) in response to different stimulation. The gray bar indicates the occurrence of stimuli. **(F)** Averaged multiunit activity in response to different stimulation. **p < 0.01, *t*-test, mean ± SEM (red: tone stimulus; blue: noise stimulus). **(G)** Averaged half-peak durations on PSTHs recorded from MECP2-TG mice and WT mice. *p < 0.05, *t*-test, ***p < 0.001, paired *t*-test, mean ± SEM (red: tone stimulus; blue: noise stimulus). **(H)** Averaged peak latency of PSTHs recorded from MECP2-TG mice and WT mice. *p < 0.05, *t*-test, mean ± SEM (red: tone stimulus; blue: noise stimulus).

Overreaction or hypersensitivity to noise is commonly observed in people with ASD (Rimland and Edelson, 1995; Kern et al., 2006; O’Connor, 2012). We compared the properties of responses to pure tones (7000 Hz) and white noise in both animal groups. At 70 dB SPL, white-noise stimulation evoked a stronger LFP than a pure tone (7000 Hz) in MECP2-TG mice but not WT mice (Figures 6A,B). The temporal profiles of LFPs, such as onset latency and peak latency, were similar in response to pure tones and white noise in both animal groups (Figures 6C,D). However, a significant delay in response was found in MECP2-TG mice compared with WT mice (Figures 6C,D). In addition to LFPs, we also investigated the MUA in the auditory cortex of both animal groups (Figure 6E). As with LFP, white noise evoked stronger responses than a pure tone at 70 dB in MECP2-TG mice (Figure 6F). For temporal profiles, the half-peak duration and peak latency of the PSTH were measured. The prolonged half-peak duration is consistent with the strengthened response to noise stimulation (Figure 6G). The peak latency in response to noise stimulation was significantly increased (Figure 6H).

**FIGURE 7:**
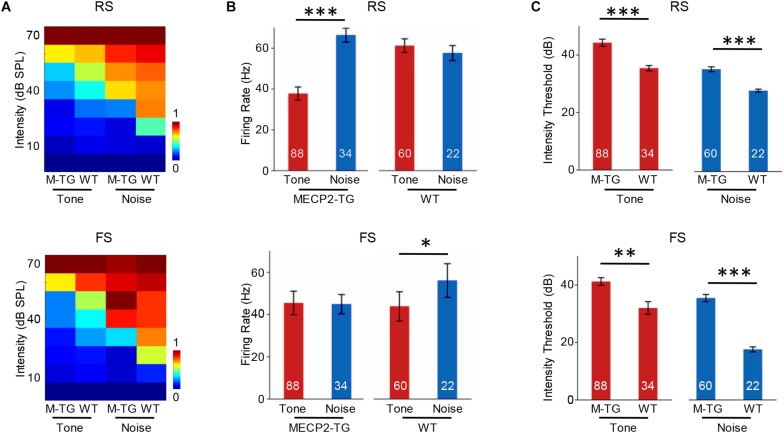
**(A)** Average receptive fields of regular-spiking (RS) and fast-spiking (FS) neurons recorded from MECP2-TG and wild-type (WT) mice in response to a pure tone [7000 Hz, 70 dB sound pressure level (SPL)] and white noise (70 dB SPL). **(B)** Averaged activity of RS and FS neurons in response to a pure tone (7000 Hz, 70 dB SPL) and white noise (70 dB SPL) at 70 dB. *p < 0.05, ***p < 0.001; paired *t*-test; mean ± SEM. **(C)** Average intensity threshold of RS and FS neurons recorded from MECP2-TG mice and WT mice to a pure tone and white noise. **p < 0.01, ***p < 0.001, paired *t*-test, mean ± SEM (red: tone stimulus; blue: noise stimulus).

We then recorded 88 RS and 34 FS neurons from MECP2-TG mice as well as 60 RS neurons and 22 FS neurons from WT mice using single-cell recording. Figure 7A shows the intensity-dependent tuning of normalized responses to a pure tone (7000 Hz) and white noise. For RS neurons, MECP2-TG mice exhibited a stronger response to white noise than to the pure tone. For FS neurons, WT mice showed stronger responses to white noise than to the pure tone (Figure 7B). These comparisons suggest a potential lack of cortical inhibition when MECP2-TG mice were exposed to a noisy environment. Meanwhile, the thresholds of both RS and FS neurons were higher in MECP2-TG mice than in WT mice (Figure 7C), which is consistent with the findings from the cortical threshold map (Figure 2).

## Discussion

Previous studies have reported that individuals with RTT (loss of MECP2) have normal ABRs but delayed auditory cortex responses (Oram Cardy et al., 2005; Cardy et al., 2008; Russo et al., 2009; Roberts et al., 2011). In this study, we found that MECP2 overexpression did not strongly affect the major properties of ABRs, such as threshold and peak latencies. These results suggested that there was no major difference between MECP2-overexpressing animal and their WT littermates at the early stage of the auditory system. However, a significant increase in threshold was found in the cortical audiogram, suggesting that the processing of auditory information at the cortical level could be different. Thus, a hearing abnormality could gradually arise from MECP2 overexpression in the ascending auditory pathway. We then focused on the properties of cortical neurons in both genotypes. Analysis and comparison of spikes revealed similar waveforms from RS cells but not FS cells. The altered spike waveform of FS cells indicates that aberrant intracellular dynamics were induced by MECP2 overexpression (e.g., composition of ion channels). These results suggest that MECP2 overexpression might not completely silence the activity of a neuron but could gradually change the transmission of information in a multiple-step circuit such as those in the visual, auditory and somatosensory ascending pathways.

Previous studies have found that individuals with ASD often have difficulty understanding speech in noisy environments (Alcantara et al., 2004; Mamashli et al., 2016). In this study, we found that the responses evoked by noise were stronger than those evoked by tone stimulation in MECP2-TG mice. In addition, we found that MECP2-TG mice have a higher intensity threshold than WT mice. These findings suggest that MECP2-TG mice might have difficulty hearing clearly in a noisy environment. In other words, the perception of noise could overwhelm the ability of the animal to perceive the frequency features of sound. This phenotype is potentially related to the noise hypersensitivity commonly found in people with ASD. However, this hypothesis needs to be tested with behavioral evidence and neuron manipulation approaches such as optogenetics.

In addition to noise sensitivity, another interesting finding of this study is that many atypical responses in MECP2-TG mice are directly related to FS neurons, which largely overlap with parvalbumin inhibitory neurons (Li et al., 2015). The excitation/inhibition (E/I) balance plays an important role in the physiology of the nervous system. Increased E/I in key neural systems has long been considered a critical cause of autism-related abnormalities (Rubenstein and Merzenich, 2010), and inhibitory circuits are critical for adjusting the E/I balance. A disrupted E/I balance in a mouse model of ASD can cause sensory abnormalities such as tactile hypersensitivity (Orefice et al., 2016). Goffin and his colleagues (Darren et al., 2014) demonstrated that the appropriate level of MECP2 in GABAergic neurons was crucial for auditory information processing, and the preservation of MECP2 function in GABAergic neurons can restore auditory processing in MECP2-null mice. Thus, inhibitory neurons might also cause auditory abnormalities and play a critical role in the atypical responses observed in MECP2-TG mice. The narrowed tuning bandwidth of FS neurons might result in a lack of inhibition, which could be related to the unusual noise sensitivity found in MECP2-TG mice. In summary, our study revealed that many fundamental response properties of the auditory cortex in MECP2-TG mice are atypical compared with those of WT littermates, including latency, duration, spike waveform, firing rate, and firing threshold; this finding could be helpful for further understanding auditory deficits in autism.

## Ethics Statement

This study was carried out in accordance with the recommendations of Animal Care and Use Committee of Army Medical University. The protocol was approved by the Animal Care and Use Committee of Army Medical University.

## Author Contributions

YZ and YX designed and supervised the experiments. CZ and SY performed most of the experiments. SQ, ZW, and ZS assisted the work. CZ and YZ wrote the manuscript.

## Conflict of Interest Statement

The authors declare that the research was conducted in the absence of any commercial or financial relationships that could be construed as a potential conflict of interest.
