# Supplementary material for: Atypical Response Properties of the Auditory Cortex of Awake MECP2-Overexpressing Mice
Source: Front Neurosci. 2019 May 7;13:439. doi: 10.3389/fnins.2019.00439 (PMC6515258; doi:10.3389/fnins.2019.00439)
Supplement: Supplementary file 1 [file Table_1.DOC]

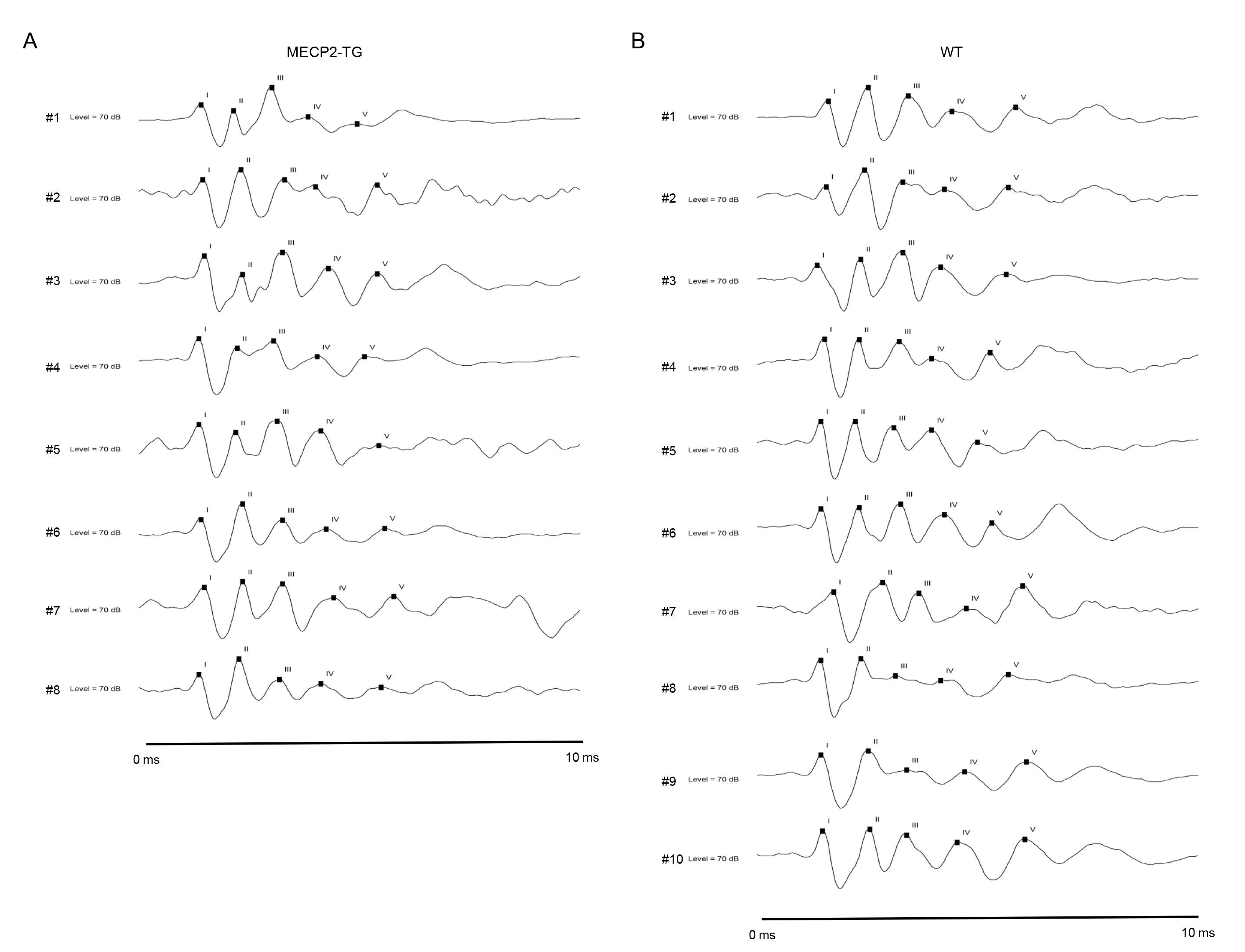


Fig. S1

A. Raw traces of auditory brainstem responses (ABRs) at 70dB SPL from eight MECP2-TG mice (#1 to #8).

B. Raw traces of ABRs at 70dB SPL from ten WT mice (#1 to #10).

Black squares indicate the locations of peak I - V.


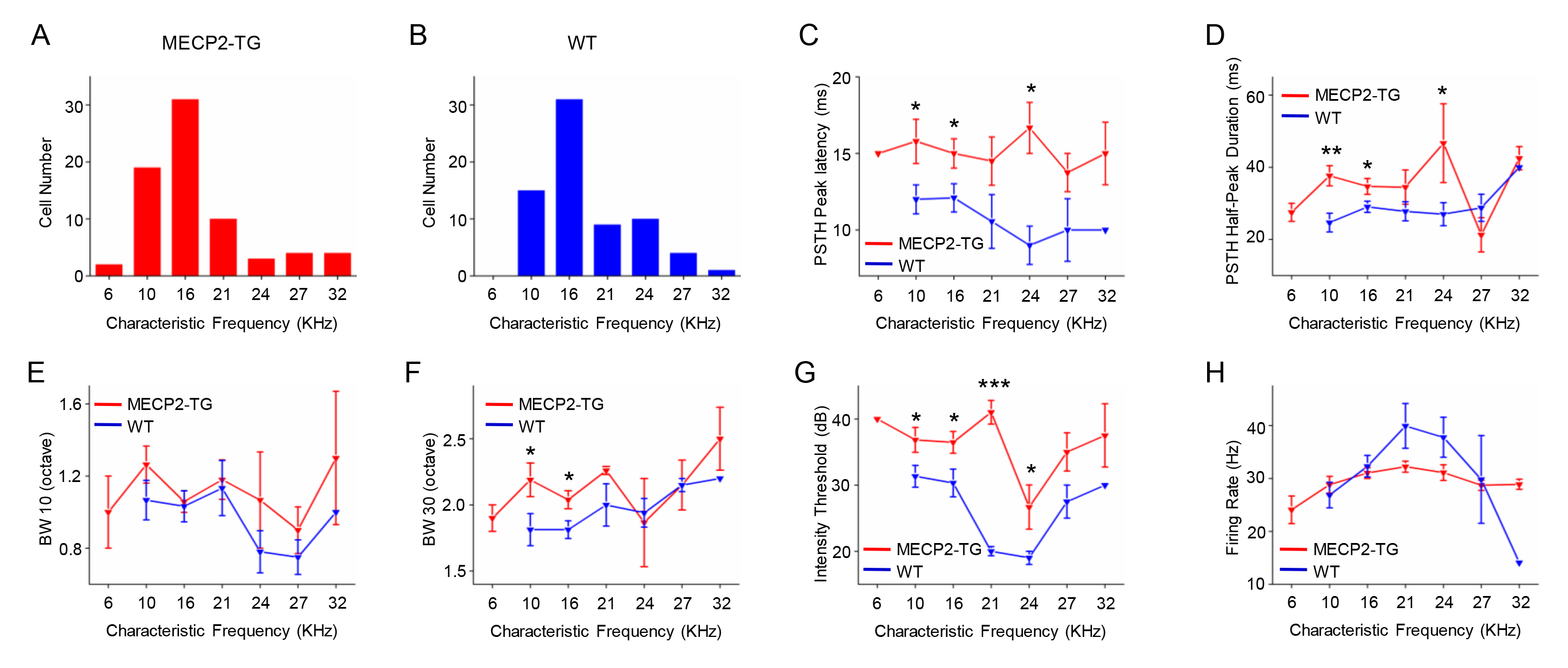


Fig. S2

A. Distribution of characteristic frequency of RS neurons (n = 73) recorded from MECP2-TG mice.

B. Distribution of characteristic frequency of RS neurons (n = 70) recorded from WT mice.

C. Averaged peak latency of RS neurons with different CF recorded from MECP2-TG mice and WT mice, [respectively](javascript:void(0);). *, p<0.05, t-test, mean ± standard error of the mean (SEM).

D. Averaged duration of PSTH measured at the half-maximum level of RS neurons in different CF recorded from MECP2-TG mice and WT mice, [respectively](javascript:void(0);). *, p<0.05, **, p<0.01, t-test, mean ± SEM.

E & F. Average tuning bandwidth of TRF at 10 dB and 30 dB above the intensity threshold of RS neurons in different CF recorded from MECP2-TG mice and WT mice, [respectively](javascript:void(0);). *, p<0.05, t-test, mean ± SEM.

G. Average intensity threshold at CF of RS neurons in different CF recorded from MECP2-TG mice and WT mice, [respectively](javascript:void(0);). *, p<0.05, ***, p<0.001, t-test, mean ± SEM.

H. Averaged activity of RS neurons to BF ± 0.2 octave at 70dB in different CF recorded from MECP2-TG mice and WT mice, [respectively](javascript:void(0);). t-test, mean ± SEM.


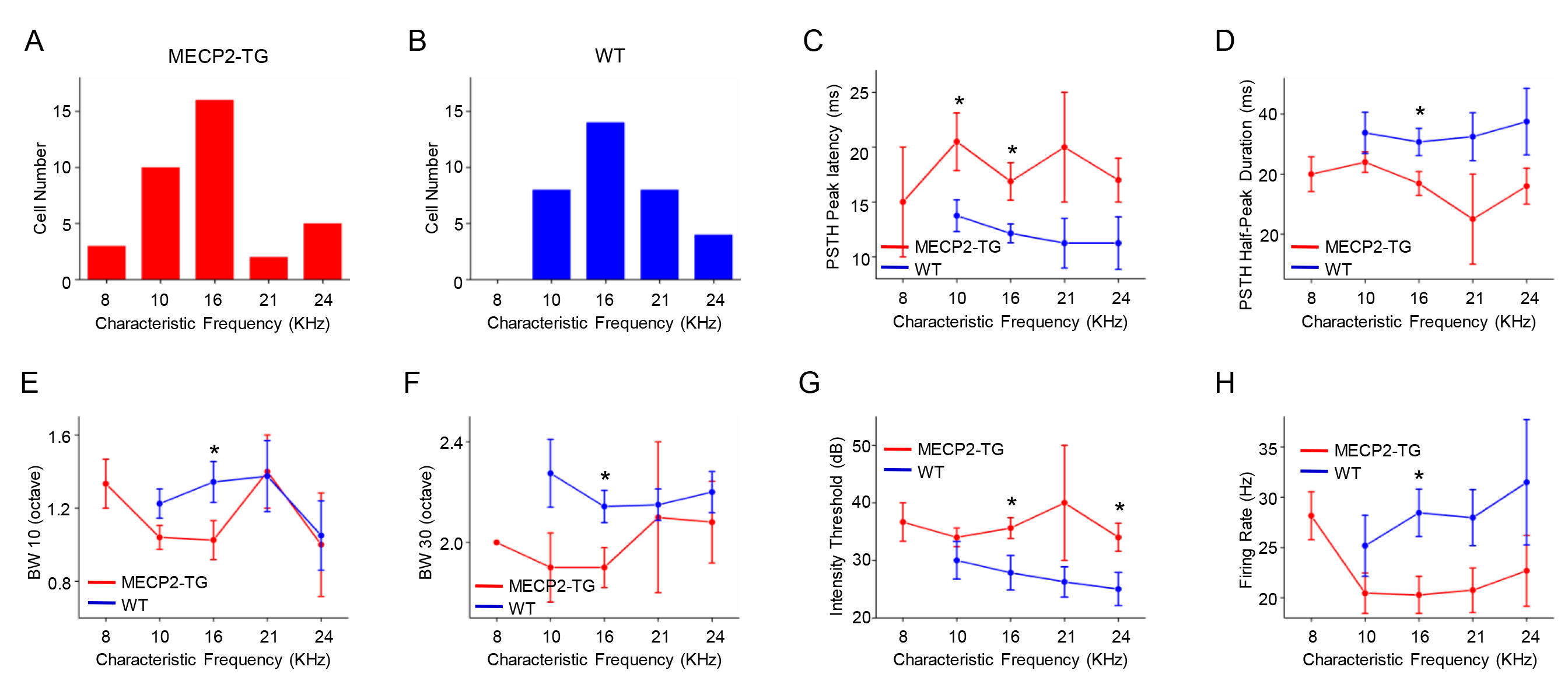


Fig. S3

A. Distribution of characteristic frequency of FS neurons (n = 36) recorded from MECP2-TG mice.

B. Distribution of characteristic frequency of FS neurons (n = 30) recorded from WT mice.

C. Averaged peak latency of FS neurons in different CF recorded from MECP2-TG mice and WT mice, [respectively](javascript:void(0);). *, p<0.05, t-test, mean ± standard error of the mean (SEM).

D. Averaged duration of PSTH measured at the half-maximum level of FS neurons in different CF recorded from MECP2-TG mice and WT mice, [respectively](javascript:void(0);). *, p<0.05, t-test, mean ± SEM.

E & F. Average tuning bandwidth of TRF at 10 dB and 30 dB above the intensity threshold of FS neurons in different CF recorded from MECP2-TG mice and WT mice, [respectively](javascript:void(0);). *, p<0.05, t-test, mean ± SEM.

G. Average intensity threshold at CF of FS neurons in different CF recorded from MECP2-TG mice and WT mice, [respectively](javascript:void(0);). *, p<0.05, t-test, mean ± SEM.

H. Averaged activity of FS neurons to BF ± 0.2 octave at 70dB in different CF recorded from MECP2-TG mice and WT mice, [respectively](javascript:void(0);). *, p<0.05, t-test, mean ± SEM.
